# Body composition and anaerobic power: correlational analysis in male athletes

**DOI:** 10.3389/fspor.2025.1666296

**Published:** 2025-11-13

**Authors:** Hebah Ali, Razan Omoush, Adam Amawi, Hadeel Ghazzawi

**Affiliations:** 1 Department of Nutrition and Food Technology, School of Agriculture, The University of Jordan, Amman, Jordan; 2 Department of Movement Sciences and Sports Training, School of Sport Sciences, The University of Jordan, Amman, Jordan

**Keywords:** body composition, muscle mass, visceral fat, anaerobic performance, wingate test

## Abstract

**Background:**

Body composition monitoring is vital to improve functional performance outcomes such as power output and fatigue resistance in athletes.

**Objective:**

To assess the correlation between body composition parameters (body fat percentage, percentage of muscle mass, and visceral fat VF) and anaerobic performance measures, specifically relative peak power (RPP) and fatigue index (FIWAnT), in male athletes. Along with exploring the potential influence of sport type, training frequency and smoking status.

**Methods:**

A cross-sectional study of 31 healthy male athletes aged 18–35 years was conducted. Participants were categorized by weekly training frequency and sport type. Bioelectrical Impedance Analysis was used to assess Body composition, and the 30 s Wingate test for Anaerobic performance.

**Results:**

A significant positive correlation was found between muscle percentage and both RPP (*r* = 0.51, *p* < 0.01) and average RPP (*r* = 0.47, *p* < 0.01). A significant negative correlation was found between average RPP and both Fat percentage (*r* = −0.45, *p* < 0.05) and VF (*r* = −0.50, *p* < 0.05). No significant correlation was found between FIWAnT and any body composition measures.

**Conclusion:**

Body composition has a critical role in the integrity of anaerobic performance among athletes.

## 1 Introduction

The performance of athletes is multifactorial, including strength, endurance, and recovery capacity; modulated by body composition and fitness level (1). Beyond training, current research increasingly explores how various lifestyle habits modulate body composition and anaerobic outcomes. Factors like chronic stress, poor sleep quality, nutritional deficiencies, and tobacco use are known to alter hormonal balance, increase inflammation, and negatively affect muscle repair and body composition (2), further impacting anaerobic capacity.

**Figure 1:**
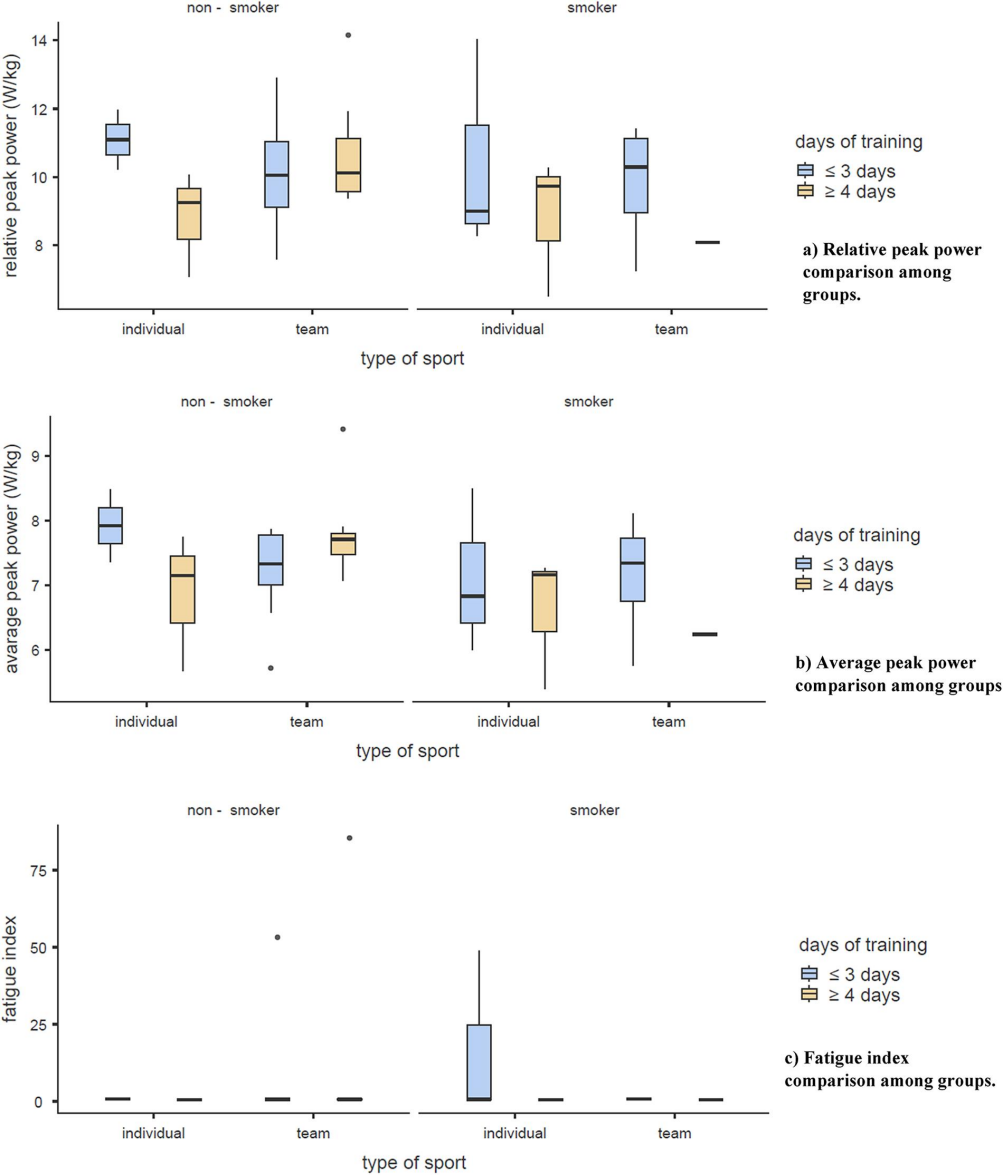
Presents WAnT outcomes [**(a)** RPP, **(b)** average RPP, **(c)** fatigue index] broken down by type of sport, frequency of training, and smoking status. As previously reported, no statistically significant differences were observed across any comparison.

Body composition in athletes, like muscle mass (MM) and fat mass (FM), needs to be monitored to improve functional outcomes such as power output and fatigue resistance (3, 4). While the general influence of FM is known, recent international studies highlight the specific role of VF accumulation, even in athletes. VF is highly metabolically active and is linked to chronic low-grade inflammation, which can potentially impair muscle function, metabolic efficiency, and recovery, thereby negatively impacting power-to-mass ratio (5).

Bioelectrical Impedance Analysis (BIA) is a practical, validated tool to assess the parameters of body composition in athletes (6), measuring estimates for muscle and fat percentage, and Visceral Fat (VF) with high convenience and reliability (7), BIA has been used effectively to track seasonal changes and training adaptations in athletes, presenting strong associations with the outcomes of performance (8).

The impact of consistent structured training on anaerobic performance is well-documented. Regular training enhances both the capacity to generate high power and the ability to tolerate fatigue, primarily by increasing glycolytic enzyme activity, buffering capacity, and the size of fast-twitch muscle fibers (9). The Wingate Anaerobic Test (WAnT) is commonly recognized to evaluate high-intensity, short-duration performance; It measures peak anaerobic power and fatigue index (FIWAnT) (10). It is used across diverse athletic populations to assess explosive strength and fatigue resistance (11). Anaerobic power reflects the capacity to perform high-intensity, short-duration work relying primarily on the phosphocreatine (PCr) and glycolytic systems (12). It appears to be sensitive to body composition, with prior studies suggesting that lean MM is positively associated with power output, while high FM often has a negative association (13).

Studies have examined the role of strength training programs on anaerobic power in specific team sports, such as volleyball (9), demonstrating training-specific adaptation. Concurrently, other research has highlighted the relationship between specific body composition profile and high-level performance in highly specialized sports, such as elite motorcycle speedway riders (14).

In addition to those physiological components, the performance of athletes is also modulated by several aspects of their current lifestyle and habits, including diet, physical activity, sleep, and factors such as tobacco use (2, 15). Smoking is often overlooked in athletic research, although it has been associated with impaired cardiovascular and pulmonary function, which may affect anaerobic capacity and fatigue levels (16). Recent systematic reviews confirm that nicotine dependence and withdrawal can negatively influence exercise-related physical ability and sport performance, underscoring the importance of considering smoking status as a potential confounding factor in athletic studies (17). Also, anaerobic output was improved after regular training by increasing endurance and muscular strength (18). The social relevance of this investigation lies in informing targeted training and nutritional strategies to enhance power and sports health monitoring in athletes (19).

As sports science evolves, understanding how detailed body composition and daily habits affect the performance metrics is important. While the correlation between lean mass and power is established, the integrated influence of specific adverse factors, visceral fat, and chronic lifestyle factors like smoking, on anaerobic performance in a homogenous group of trained male athletes remains poorly defined in the current literature. Furthermore, more determinants of FIWAnT distinct from peak power require further investigation using morphological metrics. Therefore, the aim of this study was to evaluate the relationship between body composition parameters (fat percentage, muscle percentage, and VF) and anaerobic performance; relative peak power (RPP) and FIWAnT, in male athletes, while also exploring the potential influence of smoking status, sport type and training frequency. Based on current literature, we hypothesized that RPP would be positively correlated with body fat percentage and VF levels. Also, the lack of significance in anaerobic performance would be observed across categories due to a homogenous profile of the trained sample.

## 2 Methodology

### 2.1 Study design and participants

This observational cross-sectional study was conducted of 31 male athletes aged between 18 and 35 years to investigate how anaerobic performance is influenced by body composition. Participants were recruited from training centers and various sports clubs in Amman, Jordan, through social media outreach and direct contact. To minimize bias, consistent recruitment messaging was used, emphasizing the study's focus on trained athletes. Participants were included according to the criteria: apparently healthy and had a minimum of two years of consistent training experience. The health status was determined based on self-reported information, specifically the absence of any chronic diseases or medication use.

Exclusion criteria included: the presence of any chronic disease, current use of medication, and female sex. Interested individuals were briefed on the study's objectives and procedures and were asked to sign informed consent forms before data collection. A formal sample size calculation was not performed to determine the minimum required number of participants. However, a *post-hoc* power analysis was conducted using G*power software (version 3.1.9.4) based on the strongest findings: the correlation between RPP and percentage of muscle mass (*r* = 0.51). Assuming an alpha level of 0.05 (two-tailed) and a sample size of *n* = 31, the achieved statistical power for this effect was calculated to be power = 0.89. Conversely, for smaller effects (e.g., *r* = 0.30), the achieved power drops below the conventional 0.80 threshold. This calculation confirms that while the study was adequately powered to detect large effects, it was underpowered to detect small or moderate correlations, increasing the likelihood of type II errors in non-significant findings.

Consequently, the study relies on a convenient sample of 31 athletes. We acknowledge that this limited sample size may have resulted in low statistical power, particularly for detecting moderate effects or differences in group comparison. The absence of this power analysis is a recognized methodological limitation of this study.

### 2.2 Sport classification

Athletes were categorized to better understand the differences in sport-specific demands and training load; Training Frequency (the number of training days per week: ≤4 days/week and >4 days/week). And sport Type in which participants were classified into two broad categories: team and individual sports. This approach was used to compare general differences in chronic demand. However, the lack of a more specific classification limits the analysis by potentially masking obvious differences between distinct physiological phenotypes. The intensity of training commitment and the nature of the sport were factored into the study the possibility of potential differences in body composition and anaerobic performance related to them.

### 2.3 Body composition assessment

Body composition was assessed using the InBody 770 (Inbody Co., LTD, Seoul, Korea; Frequencies: 1, 5, 250, 500, and 1,000 kHz) multi-frequency segmental BIA device (20). Participants were requested to fast for at least 4 h prior to the assessment. Weight, FM, and percentage, MM, and percentage, and VF levels were provided by the BIA assessment.

### 2.4 WAnT

Following the BIA assessment, participants consumed a light snack (plain biscuit and water) and were asked if they were well rested before performing the WAnT. They were asked to wear comfortable clothing. The test was conducted on a cycle ergometer (Monark 894E, Sweden) connected to a computer system for accurate monitoring, in which the test was started with a simple warm-up of light pedaling for 10 min (21). Then, each athlete was instructed to pedal for 30 s at maximal effort against a 7.5% kg of body weight resistance. The task was to perform work to achieve maximal power with a quick rotation frequency and try to maintain it as long as possible. Encouragement was provided by verbal motivation to reach maximal effort. After the test, the subject remained on the cycle ergometer for 5 min for safety reasons (22). The test yielded values for maximum peak power, RPP, average peak power, average RPP, and FIWAnT.

### 2.5 Smoking status

Participants were asked if they were smoking any type, including cigarettes, shisha, or electronic devices like vapes, and recorded it using a self-reported questionnaire.

### 2.6 Statistical analysis

All statistical analyses were performed using Jamovi 2.6.26 (Computer Software, Retrieved from https://www.jamovi.org) (23). The Shapiro–Wilk test was used to check for normality of each variable. Based on that, Spearman's correlation was used for the Variables that were not normally distributed (e.g., FM, height, VF) to find the associations between body composition parameters and anaerobic performance measures. And for group comparisons, the Kruskal–Wallis test was used for the non-normally distributed to analyze WAnT outcomes. While One-way ANOVA was used to analyze FIWAnT, which was normally distributed. Three-way ANOVA was used to investigate the interaction effects between training frequency, sport type, and smoking status on FIWAnT. A significance level of *p* < 0.05 was set for all analyses.

Potential confounding factors, such as age and duration of training, were considered for statistical adjustment. However, due to the small sample size, sophisticated multivariate modeling procedures were deemed statistically inappropriate. Using complex adjustments could compromise statistical power. Instead, we focus on interpreting the primary correlational data while recognizing that these unadjusted factors constitute a study limitation.

### 2.7 Ethical approval

The Institutional Review Board at The University of Jordan (IRB at UJ) approved the research proposal submitted by Dr. Hadeel Ali Ghazzawi from the School of Agriculture, Decision No. (346/2025) The IRB at the Deanship of Scientific Research, The University of Jordan.

As participation was entirely voluntary, participants were free to exit the study at any time. Written invitations detailing the goal of the study were sent out, and each participant was requested to provide written informed consent digitally. All parts of the investigation involved the protection and anonymization of personal data. Written informed consent has been obtained from the sample participants to publish this paper.

## 3 Results

### 3.1 Descriptive statistics and normality

Data from 31 male athletes were analyzed (Table 1). Normality testing using the Shapiro–Wilk test revealed that FM, height, and VF did not follow a normal distribution (*p* < 0.05).

**Table 1:** Descriptive characteristics for the participants (*n* = 31).

Variables	*N* = (31)	
	Mean	SD
Weight (kg)	76.08	14.30
Height (cm)	171.64	24.63
Fat percentage	15.72	6.94
Muscle (kg)	35.88	5.28
Muscle percentage	48.04	4.10
RPP (W/kg)	10.23	1.93
Average RPP (W/kg)	7.38	0.95
Variables	Median	Q1,Q3
VF (cm^2^)	3	(2.0,6.5)
FM (kg)	10.3	(6.85,17.05)
FIWAnT	0.59	(0.52,0.66)

VF, visceral fat; FM, fat mass; RPP, relative peak power; FIWAnt, fatigue index.

### 3.2 Correlation analysis

As detailed in Table 2, the percentage of muscle mass demonstrated a strong, significant positive correlation with both RPP and average RPP. Conversely, body fat percentage and VF were significantly and negatively associated with power output.

**Table 2:** Spearman's correlation between body composition measurements and wingate test output.

Variables	Fat percentage	Muscle percentage	VF (cm^2^)	RPP (W/kg)	Average RPP (W/kg)
Muscle percentage	−0.990***	—			
VF (cm^2^)	0.921***	−0.895***	—		
RPP (W/kg)	−0.563***	0.573***	−0.540**	—	
Average RPP (W/kg)	−0.506**	0.530**	−0.512**	0.849***	—
FIWAnT	−0.135	0.148	−0.017	0.347	0.087

VF, visceral fat; RPP, relative peak power; FIWAnt, fatigue index.

*p < 0.05, **p < 0.01, ***p < 0.001.

**Table 3:** Group comparison between smoking, type of sport, and frequency of training.

Variables	K—1	Smoke		Sport type		Frequency	
		*H*	*p*	*H*	*p*	*H*	*p*
Fat percentage	1	0.41	0.52	0.07	0.78	0.08	0.77
Muscle percentage	1	0.64	0.42	0.05	0.81	0.08	0.77
VF (cm^2^)	1	0.27	0.60	1.80	0.17	0.90	0.34
RPP (W/kg)	1	0.43	0.50	0.11	0.73	0.47	0.49
Average RPP (W/kg)	1	1.24	0.26	0.93	0.33	0.08	0.77
FIWAnT	1	1.06	0.30	0.22	0.63	0.20	0.65

VF, visceral fat; RPP, relative peak power; FIWAnt, fatigue index.

*p < 0.05.

**Table 4:** Interactions between groups with fatigue index FIWAnT.

Interactions	F	*p*
Days of training ✻ type of sports	0.5485	0.466
Days of training ✻ smoke	0.8135	0.376
Type of sports ✻ smoke	0.5050	0.484
Days of training ✻ type of sport ✻ smoke	5.0406	0.035*

* *p* < 0.05

### 3.3 Group comparisons

In Table 3, smoking showed no significant differences in fat percentage, muscle percentage, VF, RPP, average RPP, or FIWAnT (*p* > 0.05 for all). Sport type and training frequency also showed no statistically significant effect on body composition or Wingate outcomes (all *p* > 0.05), as shown in Figure 1.

### 3.4 FIWAnT

A three-way ANOVA was used for FIWAnT in Table 4. A statistically significant interaction effect was observed between training frequency, sport type, and smoking [F (1,23) = 5.04, *p* = 0.035], indicating that the combination of these factors influenced FIWAnT. No main effects or two-way interactions were significant on their own (*p* > 0.05).

## 4 Discussion

This study was conducted to evaluate the relationship between fat percentage, muscle percentage, and VF as the body composition variables and the WAnT indices measuring the anaerobic performance in a sample of male athletes. In addition, the influence of training frequency, sport type, and smoking status on body composition and performance was assessed. The primary findings of this investigation are summarized as follows: i) a statistically significant positive correlation exists between the percentage of muscle mass and the RPP. ii) body fat percentage and VF exhibit a significant negative correlation with average RPP. iii) No significant correlation was found between any body composition variables and the FIWAnT, nor were there significant differences across training or lifestyle groups.

One of the significant findings was the strong negative correlation between fat percentage and both RPP and average RPP, suggesting that reduced anaerobic capacity is influenced by increased body fat. Fat percentage has different effects regarding athletes, as supported by previous literature, performance and metabolic flexibility. The excess FM can diminish performance and act as a non-functional weight in high-intensity, short-duration effort like the WAnT (24–26). Metabolic flexibility, where the ability of the body to switch between carbohydrate and fat as a fuel is impaired, which leads to a compromised exercise capacity (27).

To the contrary, muscle percentage correlated positively with both power measures, emphasizing the importance of MM in supporting anaerobic yield. This is consistent with the well-established understanding of the physiology of MM being responsible directly for providing the force required during anaerobic strength (26, 28–30). Particularly fast-twitch fibers, which can supply more power and energy for short, intense activity (31). Moreover, MM contributes to better performance, not only by the composition of muscle (32), which means increased MM will facilitate energy without oxygen more rapidly through the breakdown of glucose and phosphocreatine (30).

Different correlations between body composition variables were found, and they may collectively influence anaerobic performance. For instance, the strong inverse correlation between fat percentage and muscle percentage is expected, and the strong positive correlation between fat percentage and VF suggests that an increase in overall body fat is linked to higher accumulation of fat around the internal organs, which has been shown to be detrimental to performance (33).

A negative correlation was found between FIWAnT and RPP and average RPP, which indicates that the accumulation of fat around internal organs may compromise performance. The exact mechanisms require further investigation, but VF is often associated with metabolic disorders, which lead to a reduced availability of energy during intense performance (34). Also, the heart rate recovery after exercise is affected by the degree of VF, which delays the ability to reach the resting state efficiently (5).

Correlations between FIWAnT and any body composition indices were found to be not significant, this suggests that fatigue is rarely caused by one issue, but the result of multiple factors; such as dehydration, which impairs anaerobic performance by reducing blood volume and raising core temperature, which negatively affects muscle function and leads to a decline in power output, directly influencing FIWAnT (35). While studies have shown lean MM to be crucial for power (36), our results indicate that the subsequent decline in power FIWAnT during the 30 s WAnT is driven by processes that override the volume of muscle or FM. Also, the primary drivers of this fatigue are metabolic, specifically the rapid depletion of phosphocreatine PCr and the accumulation of lactate and hydrogen ions from glycolysis (12). Furthermore, fatigue is not just a muscle-level event; it also involves neuromuscular factors. The decline in power during a WAnT results from both peripheral fatigue at the muscle and central fatigue in the nervous system, with an individual's ability to voluntarily activate motor units being crucial for fatigue resistance (37). Therefore, the FIWAnT may be more sensitive to acute variables like hydration status (35) and training quality (38) than stable morphological measures like MM or fat percentage. This result strongly suggests the use of multivariable models in future research to assess metabolic, neuromuscular, and morphological contributions to fatigue resistance simultaneously.

No statistically significant differences in RPP, average RPP, or FIWAnT across sport type, training frequency, or smoking status in terms of group comparisons. This indicates that such factors may not substantially alter the anaerobic output within this sample of trained athletes due to the homogeneity of the sample (39). A high level of conditioning likely minimizes the physiological impact of these factors on anaerobic output. Also, the overall health of the athlete may mitigate the effects of smoking on short-term performance (40). Although a small sample size could have affected the statistical power.

However, a significant interaction was observed between smoking status, sport type, and training days on FIWAnT, suggesting that combined training behaviors and lifestyle may affect fatigue and recovery or the depletion of energy during anaerobic performance (41, 42). The type of sports, specifically team sports that usually require more anaerobic capacity, enhance the anaerobic power and the physical fitness, resulting in reduced fatigue over time (19, 43). Also, the frequency of resistance training has been shown to increase the anaerobic performance of athletes, leading to better recovery and less fatigue, by distributing the same volume of training over more sessions (18, 44). Further investigation is needed with a larger sample size to reveal more about these associations.

The absence of statistically significant differences across intergroup comparisons warrants a deeper interpretation. These null results may reflect one of two possibilities. First, it is likely a factor of the homogeneous profile of the sample; all participants were consistently trained male athletes, suggesting a ceiling effect of consistent physiological conditioning that minimized measurable differences in anaerobic capacity between groups. Second, the findings must be interpreted in light low statistical power inherent to the small sample size.

This cross-sectional study is fundamentally limited by its design, which prohibits causal inference and is susceptible to selection bias due to the homogeneous sample (young, male athletes). Methodological constraints, including reliance on self-reported smoking status and lack of strict fasting monitoring before BIA, compromise data accuracy. While our results support optimizing body composition for power, the low statistical power and limited generalizability necessitate future longitudinal studies. These studies must employ larger, diverse samples and multivariable analysis to comprehensively model the complex interplay of chronic lifestyle factors and body composition on FIWAnT.

## 5 Conclusion

Body composition has a critical role in the integrity of anaerobic performance among athletes. Specifically concerning power output. The results provide clear practical implications for training prescription and nutritional guidance in high-intensity sport. Strategies prioritize reducing FM, especially VF, while enhancing and maintaining MM to maximize RPP outputs. Furthermore, this study significantly contributes by highlighting the complexity of fatigue; the absence of correlation between body composition variables and the FIWAnT requires moving beyond univariate body metrics to fully understand fatigue resistance. While these findings are valuable, their generalizability is limited by the small sample size and the cross-sectional design. Future research should employ larger, more diverse cohorts in multivariable and longitudinal models to fully capture the combined physiological and lifestyle influence on anaerobic performance and fatigue in athletes.

## Data Availability

The raw data supporting the conclusions of this article will be made available by the authors, without undue reservation.
